# A Multi-Institutional Case Series of Neonatal Lupus Erythematosus Without Cardiac Involvement From Vietnam

**DOI:** 10.1155/crpe/5727878

**Published:** 2025-10-13

**Authors:** Cong Mai Thanh, Phuoc Nguyen Trong, Lien Luong Thi, Ha Nguyen Thi

**Affiliations:** ^1^Department of Pediatrics, Hanoi Medical University, Hanoi, Vietnam; ^2^Pediatric Center, Bach Mai Hospital, Hanoi, Vietnam; ^3^Department of Pediatrics, Hanoi Medical University Hospital, Hanoi, Vietnam

**Keywords:** lupus erythematosus, neonatal, neonatal lupus, neonatal lupus erythematosus

## Abstract

Neonatal lupus erythematosus (NLE) is a rare autoimmune disease in newborns and infants, caused by the transplacental transmission of anti-Sjögren's syndrome–related antigen A (anti-SSA), anti-Sjögren's syndrome–related antigen B (anti-SSB), and anti-ribonucleoprotein (anti-RNP) maternal antibodies to fetal tissues during pregnancy, with manifestations in the skin, heart, liver, and hematologic systems. While cardiac abnormalities in NLE, primarily congenital heart block, may be detected prenatally through fetal echocardiography, noncardiac manifestations become evident after birth and can be easily missed due to their nonspecific nature. In this report, we describe six cases of NLE without cardiac involvement, diagnosed and managed by our team across three hospitals in Hanoi, Vietnam, between 2018 and 2023, with patient ages at diagnosis ranging from 40 days to 7 months. Clinical and laboratory findings include skin rash (6/6), anemia (5/6), thrombocytopenia (3/6), neutropenia (3/6), elevated liver enzymes (2/6), hypocomplementemia (4/5), and positivity for anti-SSA (5/6), anti-SSB (4/6), and anti-RNP (2/6) antibodies. Treatment approaches consisted of intravenous immunoglobulin (IVIG) in three cases, oral corticosteroids in one case, topical corticosteroids in one case, and supportive care without specific treatment in one case. All patients demonstrated full clinical recovery without any residual sequelae.

## 1. Introduction

Neonatal lupus erythematosus (NLE) is a rare autoimmune disease affecting newborns and infants. Maternal autoantibodies, specifically anti-Sjögren's syndrome–related antigen A (anti-SSA), anti-Sjögren's syndrome–related antigen B (anti-SSB), and, less frequently, anti-ribonucleoprotein (anti-RNP), are transplacentally transferred to the fetal tissues during pregnancy, resulting in abnormalities in the skin, heart, liver, and hematological systems [1]. The incidence of NLE is approximately 0.6 per 100,000 live births annually, with an equal gender distribution [2]. Approximately 2% of infants born to mothers with anti-SSA or anti-SSB antibodies develop NLE; however, the risk of recurrence may rise to 20% in subsequent pregnancies [2].

The clinical manifestations of NLE can be categorized into reversible and irreversible features. The reversible features include cutaneous lesions and, less commonly, hepatic and hematologic abnormalities. In contrast, cardiac involvement—primarily congenital heart block—is considered irreversible. One proposed mechanism for the development of organ defects in NLE involves the exposure to SSA and SSB antigens on fetal cell surfaces during development. During the second trimester, maternal immunoglobulin G (IgG) autoantibodies cross the placenta and bind to these antigens, forming antigen–antibody complexes within the fetal organs. These complexes are then opsonized and phagocytized, triggering inflammation, cytokine release, and tissue damage [3].

Patients with NLE-related cardiac anomalies are frequently detected in utero and managed by cardiologists due to the severity and complexity of their condition. In contrast, noncardiac manifestations typically emerge postnatally with nonspecific signs and symptoms that can be overlooked or misdiagnosed, leading to inappropriate treatment. Nevertheless, noncardiac NLE cases generally have favorable prognoses, with the potential of complete recovery if appropriately diagnosed and managed. However, most recent publications focus on NLE patients with cardiac involvement or general cohorts, paying limited attention to the characteristics of noncardiac NLE cases. Therefore, in this report, we describe six cases of NLE without cardiac involvement that were diagnosed, treated, and followed up at three hospitals in Hanoi, Vietnam, between 2018 and 2023—all of which resulted in complete clinical recovery without sequelae.

## 2. Presentation of Cases

### 2.1. Case 1

**Figure 1:**
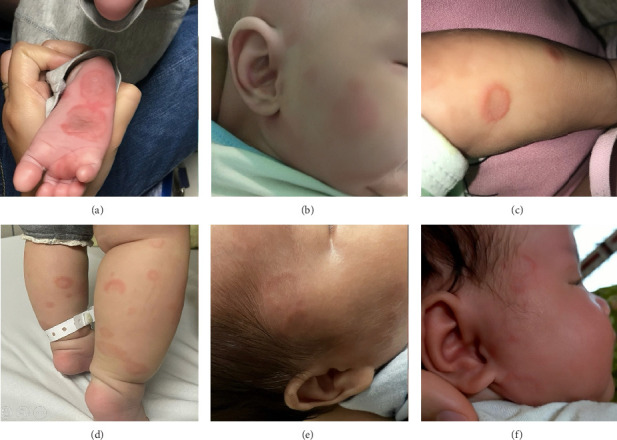
Summary of skin lesions of 6 NLE presented cases. Multiple annular erythematous rashes with central regression and raised margins on the ((a) Case 1) right sole; ((c) Case 3, (d) Case 4) legs; and ((b) Case 2, (e) Case 5, (f) Case 6) face.

A 48-day-old male infant was admitted to the Department of Immunology, Allergy, and Rheumatology, National Children's Hospital, in 2018 due to the onset of skin rashes that first appeared at 7 days of age. He was the first child, born full-term, with a birth weight of 3000 g. The rashes were distributed across both cheeks, the neck, chest, abdomen, anterior shins, and the palms and soles, with some lesions fading spontaneously while new ones continued to appear (Figure 1(a)). Further physical examination revealed no abnormalities in other organ systems, and the infant was afebrile. His mother reported experiencing similar skin lesions 2 years prior, accompanied by a positive antinuclear antibodies (ANA) test, and was diagnosed with Sweet's syndrome at the National Dermatology Hospital. Laboratory investigations revealed neutropenia with an absolute neutrophil count of 0.91 G/L. All other parameters, including hemoglobin level, platelet count, liver enzyme levels, complement component 3 (C3), and complement component (C4), were within normal limits. Electrocardiogram and echocardiogram findings were normal. Both the mother and the infant tested positive for ANA and anti-SSB antibodies, while anti-SSA and anti-RNP antibodies were negative. The infant was diagnosed with NLE and treated with topical corticosteroids for one month—hydrocortisone for facial lesions and fluocinolone for other areas. The skin lesions resolved completely after approximately 5 months, without residual scarring or hyperpigmentation.

### 2.2. Case 2

A 56-day-old male infant presented with skin rashes that began at 42 days of age at the Department of Immunology, Allergy, and Rheumatology, National Children's Hospital, in 2018. He was the first child, with no notable prenatal history. The mother was also healthy. Physical examination revealed circular, erythematous rashes with slightly raised peripheral borders and flat, lighter-colored centers, distributed on the head, face, neck, trunk, and limbs (Figure 1(b)). The remainder of the examination was unremarkable. Laboratory tests showed anemia with a hemoglobin (Hgb) level of 95 g/L, thrombocytopenia with a platelet count of 41 G/L, and a normal white blood cell count. Liver enzyme levels were elevated with glutamate oxaloacetate transaminase (GOT) at 169.1 U/L, and glutamyl pyruvate transaminase (GPT) at 175.2 U/L. Complement C3 and C4 levels were reduced (0.32 g/L and 0.01 g/L, respectively). Bilirubin levels were within normal limits, and the Coombs test was negative. Serological testing for viral infections—including hepatitis B surface antigen (HBsAg), anti-hepatitis E virus (anti-HEV), anti-hepatitis C virus (anti-HCV), anti-hepatitis A virus immunoglobulin M (HAV-IgM), cytomegalovirus immunoglobulin M (CMV-IgM), Epstein–Barr virus immunoglobulin M (EBV-IgM), and human immunodeficiency virus (HIV)—was all negative. Electrocardiogram and echocardiogram results were normal. Both the mother and the infant tested positive for ANA, anti-SSA, and anti-RNP antibodies, while the anti-SSB antibodies were negative. The diagnosis of NLE was established, and the infant was treated with intravenous immunoglobulin (IVIG) at a dose of 1 g/kg/day for two consecutive days. The skin lesions resolved completely within approximately 2 weeks, leaving no residual effects. At a follow-up visit at 3 months of age, both blood cell counts and liver enzyme levels had returned to normal.

### 2.3. Case 3

A 41-day-old male infant presented at the Department of Immunology, Allergy, and Rheumatology, National Children's Hospital, in 2020 with skin rashes that had developed at 27 days of age. He was the third-born child, delivered at full term with a birth weight of 2500 g. The mother had no significant medical history. On physical examination, circular erythematous rashes with raised borders and pale, flat centers were observed around the ears, trunk, and anterior shins (Figure 1(c)). No abnormalities were found in other organ systems. Laboratory investigations revealed cytopenia with Hgb at 88 g/L, platelet counts at 63 G/L, and neutrophil count at 0.18 G/L. C3 level was decreased at 0.56 g/L, while C4 was within normal limits (0.3 g/L). Liver enzyme and bilirubin levels were normal, and the Coombs test was negative. Electrocardiogram and echocardiogram findings were unremarkable. Tests for CMV-IgM, EBV-IgM, and HIV were all negative. Autoantibody testing in the infant showed positivity for ANA, anti-SSA, and anti-SSB antibodies, with negative results for anti-RNP and anti-dsDNA antibodies. The mother tested positive for anti-SSA and anti-SSB antibodies and negative for anti-RNP antibodies. The diagnosis of NLE was made, and the patient received a single dose of IVIG at 1 g/kg. Peripheral blood cell counts normalized within 4 days after treatment. The skin lesions remained for approximately 3 months before resolving completely.

### 2.4. Case 4

A 7-month-old female infant presented with rashes on both legs, without fever or other abnormal symptoms. She was initially examined at the Dermatology Clinic at Bach Mai Hospital in 2021, where laboratory investigations revealed anemia with a Hgb level of 75 g/L, mean corpuscular volume (MCV) of 87.5 fL, mean corpuscular hemoglobin (MCH) of 28 pg, and thrombocytopenia with a platelet count of 14 G/L. White blood cell count and C-reactive protein (CRP) were normal. Liver enzyme levels were GOT 29 U/L and GPT 9 U/L. She was then referred to the Pediatric Center for further evaluation. On physical examination, mild erythematous annular lesions with pale centers were noted primarily on both legs (Figure 1(d)). There was no subcutaneous bleeding, and the mucous membranes appeared normal. She had no recent history of vaccination or medication use, and her parents and siblings were healthy. Additional laboratory tests showed normal bilirubin levels, a negative Coombs test, negative CMV-IgM, EBV-IgM, and HIV serologies. Complement levels were decreased (C3: 0.32 g/L, C4: 0.05 g/L). Autoantibody testing revealed positive ANA, anti-SSA, and anti-RNP-70 antibodies, with negative anti-SSB antibodies. Electrocardiogram and echocardiogram results were normal. Her mother tested positive for ANA and anti-SSA antibodies, but negative for anti-SSB and anti-RNP antibodies. Red blood cell and platelet transfusions were administered, resulting in an increase in Hgb to 131 g/L and platelet count to 30 G/L. The rash gradually faded over 3 days; however, the platelet count decreased again to 15 G/L. A diagnosis of NLE was established, and the patient was treated with a single dose of IVIG at 1 g/kg. Three days after treatment, her platelet count increased to 137 × 10^9^/L.

### 2.5. Case 5

A 51-day-old male infant developed skin rashes at 20 days of age and was brought to the Pediatric Center at Bach Mai Hospital in 2023. He was the second child, delivered prematurely at 34 weeks of gestation. His mother had a history of systemic lupus erythematosus, for which she had been treated with oral methylprednisolone for 2 years and had discontinued therapy for 4 years prior to the pregnancy. During the current pregnancy, she was diagnosed with antiphospholipid syndrome and was managed with daily enoxaparin injections. On physical examination, a few annular rashes were noted on his face (Figure 1(e)). Laboratory tests showed anemia with an Hgb level of 80 g/L, MCV of 94.9 fL, and MCH of 32.5 pg. White blood cell and platelet counts were within normal limits (6.5 G/L and 304 G/L, respectively). Liver enzyme and bilirubin levels were normal, and the Coombs test was negative. C3 was decreased (0.77 g/L), while C4 was within normal range (0.12 g/L). Additionally, autoantibody testing revealed that both the infant and the mother were positive for ANA, anti-SSA, and anti-SSB antibodies and negative for the anti-RNP antibodies. Electrocardiogram and echocardiogram findings were unremarkable. The infant was diagnosed with NLE and managed conservatively with oral iron supplementation only. No specific immunomodulatory treatment was initiated. The skin lesions gradually faded and resolved completely after approximately 1 month. Hemoglobin levels increased steadily to 103 g/L at 3 months of age and 121 g/L at 5 months.

### 2.6. Case 6

A 40-day-old female infant developed skin rashes at 15 days of age, primarily on the face, with a few lesions on the body that resolved spontaneously within a few days. She was the second child, born at full term with a birth weight of 2300 g. The mother had been healthy before and during pregnancy but was diagnosed with systemic lupus erythematosus 1 month after delivery. The infant was admitted to the Pediatric Department at Hanoi Medical University Hospital in 2023. Clinical examination revealed annular erythematous lesions on the face (Figure 1(f)). Laboratory findings included anemia with an Hgb level of 95 g/L, MCV of 92.6 fL, and MCH of 30 pg. Platelet and white blood cell counts were within normal limits. Liver enzyme levels were elevated (GOT 167 U/L, GPT 148 U/L, and gamma-glutamyl transferase [GGT] 667 U/L). CRP and bilirubin levels were normal. The Coombs test was negative. Complement levels (C3 and C4) were within normal ranges. Echocardiogram and electrocardiogram results were also normal. Tests for hepatitis viruses were negative. Autoantibody testing showed positive ANA, anti-SSA, and anti-SSB antibodies and negative anti-RNP antibody. Based on clinical and laboratory features, the patient was diagnosed with NLE. Initially, she was managed with observation alone. After one month, the skin lesions had faded, and hemoglobin increased to 112 g/L; however, liver enzyme levels remained elevated (GOT 221 U/L, GPT 193 U/L). Therefore, treatment with oral prednisolone (5 mg/day) was initiated. After 1 month of therapy, liver enzyme levels normalized (GOT 55 U/L, GPT 68 U/L), and prednisolone was discontinued. The patient remained stable during 12 months of follow-up. The demographic, clinical, and laboratory characteristics of the six patients are summarized in Table 1.

## 3. Discussion

NLE was first described by Bridge and Foley in 1954, when they observed the transplacental transmission of the lupus erythematosus factor from mother to infant. That same year, McCuistion and Schoch reported the first case of NLE characterized by cutaneous lesions in an infant whose mother was later diagnosed with systemic lupus erythematosus (SLE) [2]. Although NLE is frequently observed in infants born to mothers with SLE, Sjogren's syndrome, and other autoimmune diseases, approximately 25%–60% of cases occur in infants whose mothers are asymptomatic or undiagnosed, presenting a significant diagnostic challenge [3]. In our case series, two mothers had preexisting autoimmune conditions prior to pregnancy (one diagnosed with Sweet's syndrome and one with SLE), while one mother received a postpartum diagnosis of SLE.

A neonate or infant is diagnosed with NLE when anti-SSA, anti-SSB, or anti-RNP antibodies are detected in either the mother's or infant's serum, accompanied by clinical features such as skin rashes, congenital heart block, liver damage, or hematological abnormalities [4]. In our cases, all six patients exhibited both cutaneous and hematological manifestations, while two of the six also showed evidence of hepatic involvement with varying degrees of elevated liver enzymes. In a larger and longer-term study, Kleitsch et al. found that among 190 NLE patients with cutaneous lesions, 15 (7.9%) had hepatic abnormalities, 3 (1.6%) had hematologic abnormalities, and 11 (5,8%) had both hepatological and hematologic involvement [5].

Cutaneous manifestations are observed in approximately 40% of NLE cases, whereas up to 80% of affected newborns may have normal skin at birth [6]. Unlike congenital heart block, which is relatively straightforward to diagnose, skin lesions in NLE are frequently overlooked or misdiagnosed as other dermatological conditions. These lesions in NLE typically develop within the first few weeks after birth, following exposure to sunlight [7]. Ultraviolet (UV) rays in sunlight enhance the expression of autoantigens on keratinocyte surfaces, facilitating antibody binding and subsequently triggering or exacerbating skin lesions [3]. However, UV exposure is not entirely necessary for lesion development, as approximately 20% of affected infants present with skin lesions at birth, and lesions may also appear in sun-protected areas such as the diaper region or the soles of the feet [3]. The cutaneous features of NLE resemble those of subacute cutaneous lupus erythematosus, typically presenting as elliptical or annular pink-to-red patches with fine scaling and slightly raised margins. Targetoid lesions with central pallor and discoid lesions are also common [3]. Lesions predominantly affect sun-exposed regions, with the head and face being the most frequently involved areas (95%), particularly around the periorbital region, producing the characteristic “eye mask” or “raccoon-like” appearance. Lesions may also appear around the mouth, cheeks, and temples. Less commonly, lesions are located on the trunk (25%), limbs (25%), or diffusely across the body (10%) [3]. Most cutaneous lesions are self-limiting and resolve without sequelae, though some may result in skin atrophy, pigmentary alterations, or telangiectasia, particularly in neonates [8]. In our series, skin lesions developed within the first 4 weeks of life in most cases, with the earliest onset at 7 days of age. Notably, one patient presented with skin lesions at 7 months old. All infants demonstrated classic NLE lesion characteristics—annular erythema with raised red margins and pale centers. Three patients exhibited widespread lesions involving the head, neck, trunk, and limbs; two patients (Cases 5 and 6) had lesions confined to the head and face, while one patient (Case 4) had lesions limited to the legs, which is an uncommon presentation. All skin lesions resolved completely without sequelae.

Hepatic and hematologic involvement in NLE occurs less frequently than cutaneous manifestations. Hepatic abnormalities—reported in approximately 10%–25% of cases—commonly include asymptomatic elevations in aminotransferases (GOT, GPT), hepatomegaly, and elevated GGT [3]. These findings often accompany cardiac or cutaneous manifestations but may occasionally present in isolation, complicating diagnosis. Although liver function is rarely compromised, severe dysfunction has been documented. In such cases, liver biopsy may reveal bile duct obstruction, portal fibrosis, or giant cell transformation, resembling idiopathic neonatal giant cell hepatitis [3]. In our series, two patients had mild transaminase elevations (Case 2: GOT 169.1 U/L, GPT 175.2 U/L; Case 6: GOT 167 U/L, GPT 148 U/L), without clinical signs of liver dysfunction. Enzyme levels normalized during follow-up.

In a report by Wang YA et al., among 30 NLE cases between December 1, 2008, and March 1, 2019, elevated GOT (mean 110.4 U/L, max 208 U/L) and GPT (mean 74.3 U/L, max 135 U/L) were observed in seven and five patients, respectively [9]. Hematologic abnormalities in NLE commonly include anemia, thrombocytopenia, and neutropenia and occur in approximately 10%–20% of cases. The pathogenesis is thought to involve maternal autoantibody-mediated suppression of fetal bone marrow rather than peripheral destruction of blood cells. In our cohort, anemia was the most frequent abnormality (5/6 patients), followed by thrombocytopenia and neutropenia (3/6 each). Yang et al. reported 17 cases with hematologic abnormalities in a cohort of 30 NLE patients, including 11 with anemia, 9 with thrombocytopenia, and 1 with neutropenia [4].

Autoantibody testing is essential for the diagnosis of NLE. Anti-SSA, anti-SSB, and, less commonly, anti-RNP antibodies are believed to contribute to the disease's pathogenesis. Anti-SSA (also known as anti-Ro) antibodies target two distinct antigens: Ro52 (52 kDa) and Ro60 (60 kDa). Ro52 is expressed in both the nucleus and cytoplasm, whereas Ro60 is primarily localized in the nucleus and nucleolus. Anti-SSB (anti-La) antibodies target a 48-kDa nuclear protein (La antigen). Anti-RNP antibodies bind to proteins A and C, which are integral to small nuclear RNA (snRNA) and play a key role in pre-messenger RNA (pre-mRNA) processing [3]. The type and titer of autoantibodies in maternal or neonatal serum can influence clinical presentation. A patient may test positive for one or multiple antibody types.

In our series, five out of six patients were positive for two of the three antibody types. Among these, anti-SSA was the most frequently detected (5/6 patients), while anti-RNP was the least common (2/6 patients). In a report by Yang, the detection rates of anti-SSA, anti-SSB, and anti-U1RNP antibodies among 30 NLE patients were 86.7%, 33.3%, and 30%, respectively, with most patients testing positive for two types of antibodies [4]. Similarly, a systematic review by Erden et al., based on 198 studies involving 755 NLE cases, found that among patients with skin lesions, the positivity rates were 84.6% for anti-SSA, 58.9% for anti-SSB, and 11.5% for anti-U1RNP antibodies [10].

Currently, there are no universally accepted guidelines for the treatment of NLE. Management depends on the number and severity of organ systems involved, and therapeutic strategies may vary across institutions. Our approach is consistent with recommendations published by the American Academy of Pediatrics (AAP). For isolated cutaneous lesions, management primarily involves photoprotection and, if needed, topical corticosteroids. Systemic corticosteroids are indicated for hepatic involvement, while IVIG at 1 g/kg/day for 1–2 days is recommended in cases of significant thrombocytopenia or anemia [3, 11]. In our series, three patients (Cases 2, 3, and 4) received IVIG for thrombocytopenia; one patient (Case 6) was treated with oral corticosteroids for persistent liver enzyme elevation; one patient (Case 1) received topical corticosteroids for extensive skin lesions; and one patient (Case 5) was managed conservatively without medication due to mild symptoms. All six patients responded well to treatment, achieved full recovery, and exhibited no long-term sequelae.

The prognosis of NLE largely depends on the organ systems involved. As maternal autoantibodies gradually decline, most cases with cutaneous, hepatic, hematologic, or neurologic manifestations resolve spontaneously within 6–8 months. In contrast, cardiac manifestations—particularly congenital heart block—are typically irreversible and associated with a higher mortality rate [3, 12]. In our series, the oldest infant diagnosed with NLE was 7 months old. This highlights the importance of maintaining clinical vigilance for NLE even beyond the neonatal period when signs are suggestive.

This case series was conducted at three pediatric centers in Hanoi, Vietnam: the Department of Immunology, Allergy, and Rheumatology of National Children's Hospital, the Pediatric Center of Bach Mai Hospital, and the Pediatric Department of Hanoi Medical University Hospital, during the period from 2018 to 2023.

## 4. Conclusion

NLE is a rare autoimmune condition affecting newborns and infants. Its manifestations primarily occur postnatally, except for congenital heart block, which may be detected prenatally through fetal echocardiography. Noncardiac presentations of NLE are typically associated with a favorable prognosis and often resolve spontaneously as maternally derived autoantibodies wane. Nonetheless, it is essential for pediatricians and dermatologists to maintain a high index of suspicion for extra-cardiac NLE in order to facilitate timely diagnosis and initiate appropriate management.

## Figures and Tables

**Table 1:** Summary of characteristics of six presented NLE cases.

Characteristics	Case 1	Case 2	Case 3	Case 4	Case 5	Case 6
Gender	Male	Male	Male	Female	Male	Female
Age at diagnosis	48 days	56 days	41 days	7 months	51 days	40 days
Birth weight (grams)	3000	2900	2500	3300	1900	2300
Delivery methods	Cesarean section	Vaginal delivery	Vaginal delivery	Vaginal delivery	Cesarean section	Cesarean section
Length of hospital stay in NICU (days)	0	0	0	0	1	4
Maternal autoimmune disease	Yes (sweet's syndrome)	No	No	No	Yes (SLE)	Yes (SLE)
Skin rash	Yes	Yes	Yes	Yes	Yes	Yes
Anemia	No	Yes	Yes	Yes	Yes	Yes
Thrombocytopenia	No	Yes	Yes	Yes	No	No
Neutropenia	Yes	No	Yes	No	Yes	No
Elevated liver enzymes	No	Yes	No	No	No	Yes
C3, C4 levels	Normal	Low	Low C3	Low	Low C3	Normal
Maternal anti-SSA antibodies	−	+	+	+	+	N/A
Neonatal anti-SSA antibodies	−	+	+	+	+	+
Maternal anti-SSB antibodies	+	−	+	−	+	N/A
Neonatal anti-SSB antibodies	+	−	+	−	+	+
Maternal anti-RNP antibodies	−	+	−	+	−	N/A
Neonatal anti-RNP antibodies	−	+	−	+	−	−
Specific treatment	Topical corticosteroid	IVIG	IVIG	IVIG	None	Oral corticosteroid
Outcome	Recovered	Recovered	Recovered	Recovered	Recovered	Recovered

*Note:* IVIG: intravenous immunoglobulin; anti-SSA: anti-Sjögren's syndrome–related antigen A; anti-SSB: anti-Sjögren's syndrome–related antigen B; anti-RNP: anti-ribonucleoprotein.

Abbreviations: N/A, not available; NICU, neonatal intensive care unit; SLE, systemic lupus erythematosus.

## Data Availability

The data that support the findings of this study are available from the corresponding author upon reasonable request.
